# Evaluating controlled human malaria infection in Kenyan adults with varying degrees of prior exposure to *Plasmodium falciparum* using sporozoites administered by intramuscular injection

**DOI:** 10.3389/fmicb.2014.00686

**Published:** 2014-12-12

**Authors:** Susanne H. Hodgson, Elizabeth Juma, Amina Salim, Charles Magiri, Domtila Kimani, Daniel Njenga, Alfred Muia, Andrew O. Cole, Caroline Ogwang, Ken Awuondo, Brett Lowe, Marianne Munene, Peter F. Billingsley, Eric R. James, Anusha Gunasekera, B. Kim L. Sim, Patricia Njuguna, Thomas W. Rampling, Adam Richman, Yonas Abebe, Gathoni Kamuyu, Michelle Muthui, Sean C. Elias, Sassy Molyneux, Stephen Gerry, Alex Macharia, Thomas N. Williams, Peter C. Bull, Adrian V. S. Hill, Faith H. Osier, Simon J. Draper, Philip Bejon, Stephen L. Hoffman, Bernhards Ogutu, Kevin Marsh

**Affiliations:** ^1^The Jenner Institute, University of OxfordOxford, UK; ^2^Centre for Clinical Research, Kenya Medical Research InstituteNairobi, Kenya; ^3^Centre for Research in Therapeutic Sciences, Strathmore UniversityNairobi, Kenya; ^4^Centre for Geographical Medical Research (Coast), Kenya Medical Research Institute - Wellcome TrustKilifi, Kenya; ^5^Sanaria Inc.Rockville, MD, USA; ^6^Centre for Statistics in Medicine, University of OxfordOxford, UK; ^7^Department of Medicine, Imperial College LondonLondon, UK

**Keywords:** malaria, challenge, falciparum, immunity, CHMI

## Abstract

**Background:** Controlled human malaria infection (CHMI) studies are a vital tool to accelerate vaccine and drug development. As CHMI trials are performed in a controlled environment, they allow unprecedented, detailed evaluation of parasite growth dynamics (PGD) and immunological responses. However, CHMI studies have not been routinely performed in malaria-endemic countries or used to investigate mechanisms of naturally-acquired immunity (NAI) to *Plasmodium falciparum*.

**Methods:** We conducted an open-label, randomized CHMI pilot-study using aseptic, cryopreserved *P. falciparum* sporozoites (PfSPZ Challenge) to evaluate safety, infectivity and PGD in Kenyan adults with low to moderate prior exposure to *P. falciparum* (Pan African Clinical Trial Registry: PACTR20121100033272).

**Results:** All participants developed blood-stage infection confirmed by quantitative polymerase chain reaction (qPCR). However one volunteer (110) remained asymptomatic and blood-film negative until day 21 post-injection of PfSPZ Challenge. This volunteer had a reduced parasite multiplication rate (PMR) (1.3) in comparison to the other 27 volunteers (median 11.1). A significant correlation was seen between PMR and screening anti-schizont Enzyme Linked Immunosorbent Assays (ELISA) OD (*p* = 0.044, *R* = −0.384) but not when volunteer 110 was excluded from the analysis (*p* = 0.112, *R* = −0.313).

**Conclusions:** PfSPZ Challenge is safe and infectious in malaria-endemic populations and could be used to assess the efficacy of malaria vaccines and drugs in African populations. Whilst our findings are limited by sample size, our pilot study has demonstrated for the first time that NAI may impact on PMR post-CHMI in a detectable fashion, an important finding that should be evaluated in further CHMI studies.

## Introduction

Controlled human malaria infection (CHMI) studies, where healthy volunteers are infected with *P. falciparum* to assess the efficacy of novel malaria vaccines and drugs, have become a vital tool to accelerate vaccine and drug development (Chulay et al., 1986; Mccarthy et al., 2011; Sauerwein et al., 2011; Roestenberg et al., 2012a). As CHMI trials are carried out in a controlled environment, they allow unprecedented detailed evaluation of parasite growth dynamics (PGD) and immunological responses to infection. However, to date they have not been used to help understand the mechanisms and impact of naturally-acquired immunity (NAI) to *P. falciparum* infection (Sheehy et al., 2013a).

Repeated infection with *P. falciparum* leads to the development of NAI, conferring protection in adults against severe disease (White et al., 2014). Whilst antibodies against blood-stage antigens are thought to be key mediators of natural immunity, the exact mechanism(s) of protection in humans *in vivo* remain unknown (Langhorne et al., 2008). Evidence from a comparison of parasite multiplication rates (PMR) showed a 7-fold reduction in PMRs between malaria-naïve volunteers in CHMI studies in Oxford vs. Gambian adults with NAI, suggesting that NAI impacts on PMR in a detectable fashion that could be quantified in a modern CHMI study (Douglas et al., 2011). It should therefore be possible to correlate prior exposure to malaria (a generally accepted surrogate of natural immunity) (Langhorne et al., 2008) with PMR following CHMI, and in turn seek to examine potential correlates of NAI. As well as identifying important antigenic targets, this could allow validation of proposed *in vitro* measures of NAI, including growth inhibition antibody activity (GIA) and antibody-dependant cellular assays (Tippett et al., 2007; Joos et al., 2010; Duncan et al., 2012; Hill et al., 2012; Sheehy et al., 2013a).

Though commonly performed in malaria-naïve populations (Sauerwein et al., 2011), CHMI trials have rarely been conducted in malaria endemic regions (Sheehy et al., 2013a). In 1957 in Lagos, 22 adults with prior exposure to malaria were challenged with “*an intravenous injection of blood heavily parasitized with P. falciparum*” (Bruce-Chwatt, 1963a,b). Whilst infection was successfully induced in 11 volunteers, examination of parasite load measured by blood film from day of injection of parasites for up to 8 weeks clearly showed individuals controlling and in some cases, clearing parasitemia. In a study published in 1954 conducted in Nairobi 30 adults were challenged with *P. falciparum* malaria (Allison, 1954). The study clearly demonstrated a protective effect of sickle cell trait in the 15 individuals with this condition. A further study involving the inoculation of semi-immune individuals with *P. falciparum* was also conducted in Liberia in the 1960s (Bray et al., 1962). However, in the time since these studies, these investigations have not been repeated.

To date, the majority of recent CHMI studies have been undertaken by administration of sporozoites by mosquito-bite (Sauerwein et al., 2011; Sheehy et al., 2013a). A major obstacle to performing such studies in malaria endemic regions has been the lack of access to appropriate insectary facilities (Sheehy et al., 2013a). The development of aseptic, cryopreserved *P. falciparum* sporozoites (NF54 strain) for injection (PfSPZ Challenge) by the biotechnology company Sanaria Inc., has helped overcome this problem (Roestenberg et al., 2013; Sheehy et al., 2013b).

Three studies using PfSPZ Challenge have been published to date; two performed in malaria-naïve European volunteers and one in Tanzanian volunteers (Roestenberg et al., 2013; Sheehy et al., 2013b; Shekalaghe et al., 2014). These studies examined the effect of dose, route and volume of administration of PfSPZ Challenge on infectivity, in order to identify a protocol that reliably infects 100% of volunteers. Whilst intradermal (ID) administration was more efficient than intramuscular (IM) in terms of infectivity, 25,000 sporozoites administered IM rather than ID was the only regimen found to infect 100% of volunteers (Sheehy et al., 2013b).

We sought to establish the sporozoite CHMI model in a Kenyan setting using PfSPZ Challenge, with the aim of increasing the international capacity for efficacy testing of malaria vaccines and drugs and allowing earlier assessment of efficacy in a population for which vaccines are being developed. We enrolled 28 adults with varying degrees of prior exposure to malaria in order to include an evaluation of the effect of NAI on PGD post CHMI.

## Materials and methods (see supplementary information)

### Study design

**Figure 1:**
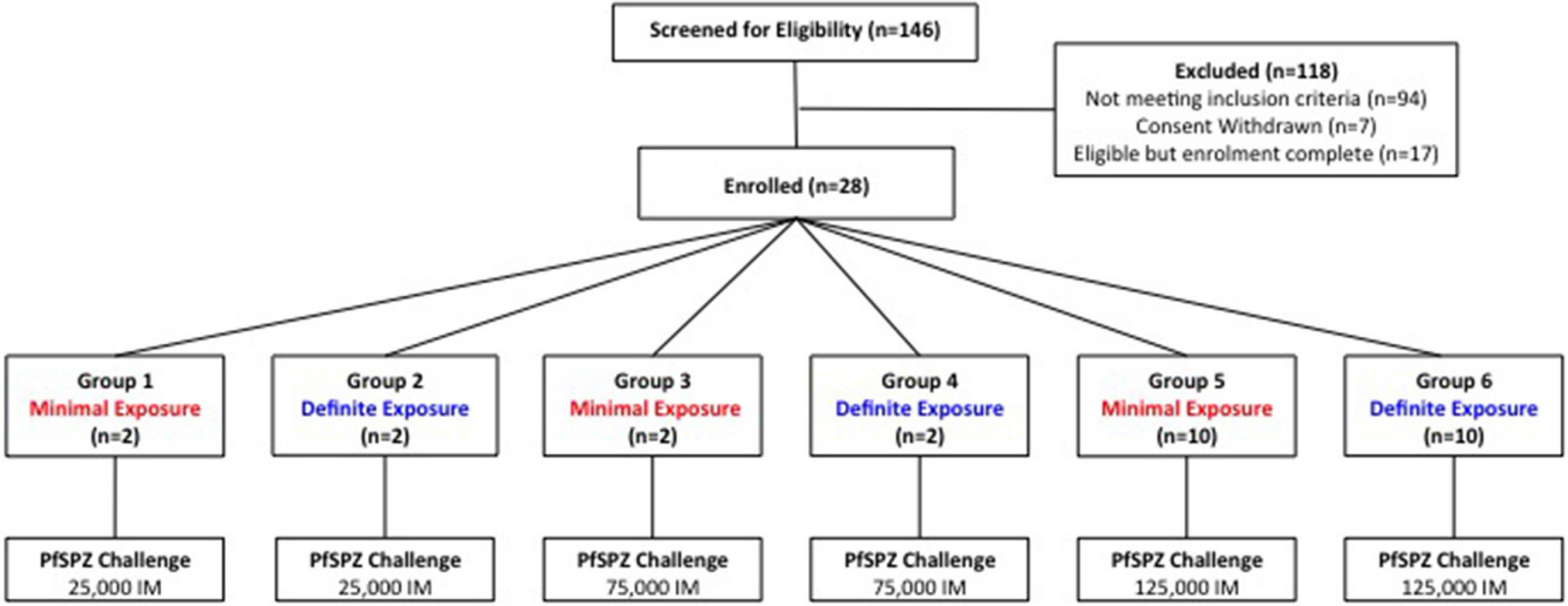
**Study design and volunteer recruitment**. 118 participants were excluded following screening (refer to Figure S1 for details). In each group, the total dose of sporozoites was split between two injection sites and administered as two 50 μL injections, one in each deltoid.

The study was an open label, randomized pilot study with blinded laboratory outcome assessment, evaluating PfSPZ Challenge administered to 28 individuals with varying degrees of prior exposure to *P. falciparum* (Figure 1). To ensure infection and detectable parasitemia post-CHMI, a dose of 125,000 sporozoites IM was chosen for the main study cohort (*n* = 20). Since the maximum dose of PfSPZ Challenge administered IM to humans at the time of study design was 25,000 IM (Sheehy et al., 2013b), two additional small cohorts receiving lower doses of 25,000 (*n* = 4) and 75,000 (*n* = 4) sporozoites IM were included in the study design to allow assessment of safety prior to administration of 125,000 sporozoites IM.

The study was conducted at the KEMRI Centre for Clinical Research, Nairobi, Kenya. Nairobi was chosen as the study site due to the lack of natural transmission of *P. falciparum* in the city, the ease of access to tertiary health care and the large number of educated adults drawn from all parts of Kenya, which would help ensure heterogeneity in prior exposure to *P. falciparum* in study participants. The study was conducted according to the principles of the Declaration of Helsinki and in accordance with Good Clinical Practice (GCP).

### Sensitization and recruitment

As the first CHMI study to take place in Kenya, the study was discussed prior to submission, in detail with the KEMRI Ethics Review Committee (ERC) and the Kenyan Ministry of Health. Following ethical and regulatory approval of the study, meetings were held with senior managers of local universities to provide information regarding the study and gain permission to hold sensitization meetings with the students at their institutions. Interested individuals at these meetings were then invited to screening appointments.

All participants were required to provide evidence of completion of secondary education (typically completed at age 16–18 years) and answer all questions on a questionnaire assessing comprehension of the study correctly prior to giving written consent. All volunteers received the study information at least 24 h in advance of the screening appointment.

### Screening

Key inclusion criteria included healthy males and non-pregnant females aged 18–40 years and agreement to stay as an in-patient from the day before administration of PfSPZ Challenge until completion of anti-malarial treatment. Key exclusion criteria included quantitative polymerase chain reaction (qPCR) positivity for *P. falciparum* at screening, hemoglobinopathies likely to impact on study results and prior receipt of an investigational malaria vaccine (see Supplementary Information for full list of inclusion and exclusion criteria).

At screening, in addition to a full medical history, examination, urinalysis, and pregnancy test in females, safety blood tests (including complete blood count, hamoglobinopathy screen, electrolytes, liver function tests and assays for HIV, hepatitis B, hepatitis C) and an electrocardiogram were performed for each volunteer to identify and exclude any individuals with baseline abnormalities (Nieman et al., 2009; Van Meer et al., 2014). Highly sensitive qPCR for *P. falciparum* was performed on screening blood samples to identify and exclude any individuals with asymptomatic parasitemia (see Supplementary Information). Volunteers were asked not to leave Nairobi between screening and enrolment in order to prevent acquisition of community-acquired *P. falciparum* infection prior to administration of PfSPZ Challenge. Volunteers positive for *P. falciparum* by qPCR at screening were treated with a therapeutic course of Co-Artem® as per national guidelines and excluded from participation. Volunteers with clinically significant illness at screening were excluded and referred for appropriate management as per national guidelines.

### Group allocation

Volunteers' prior exposure to *P. falciparum* was assessed using anti-schizont and anti-merozoite surface protein 2 (MSP2) Enzyme Linked Immunosorbent Assays (ELISA) at a serum dilution of 1:1000 as previously described (see Supplementary Information) (Osier et al., 2008). Given the lack of a known assay to reliably assess NAI to *P. falciparum* (Langhorne et al., 2008), these two antigens were chosen for screening purposes on the basis of their published positive association with prior exposure to *P. falciparum* (Marsh et al., 1989; Drakeley et al., 2005; Polley et al., 2006; Mccallum et al., 2008; Osier et al., 2008).

Absolute ELISA OD results to these two antigens for screening samples were compared to negative controls (non-exposed UK sera; *n* = 30) and the cut off for seropositivity determined as the mean absolute ELISA OD plus 3 standard deviations of the negative controls (0.23 and 0.07 for reactivity against schizont extract and MSP2 respectively). Individuals were then classified as having “minimal” prior exposure to malaria (MinExp) if both anti-schizont and anti-merozoite ELISAs were negative, and as having “definite” prior exposure to malaria (DefExp) if the anti-schizont and/or the anti-MSP2 ELISA was positive.

After exclusion of volunteers not meeting the criteria for MinExp or DefExp, 28 volunteers were identified as potential participants in the study; 14 with the highest positive anti-schizont ODs (DefExp) and 14 with negative anti-schizont and anti-MSP2 ODs (MinExp). Given the high number of participants meeting exclusion criteria (Figure S1), a number of these “first choice” DefExp individuals were unable to participate in the study. Subsequent DefExp volunteers were selected from those with the highest positive anti-schizont ELISA ODs in descending order (Absolute ELISA OD range 0.25–0.97). Once the final 28 volunteers were identified they were randomly allocated to appropriate groups (Figure 1).

### Adverse events (AEs) and criteria for treatment with anti-malarial therapy

AEs were solicited daily from injection of PfSPZ Challenge, graded for severity (according to the criteria in Tables S1, S2) and classified as definitely, probably, possibly or unlikely related to (a) PfSPZ Challenge injection, (b) malaria infection, or (c) anti-malarial therapy. Anti-malarial therapy was commenced in any one of the following circumstances; positive blood-film on microscopy; symptoms highly consistent with *P. falciparum* infection in the opinion of clinical investigators (such as fever, rigors or severe symptomatology); reaching day 21 post-injection of PfSPZ Challenge (d+C21) without blood-film positivity or symptomatology; or on withdrawal from study prior to initiation of anti-malarial therapy. A positive blood-film on microscopy was defined as at least two morphologically normal, “unambiguous” malaria parasites seen by two or more experienced microscopists blinded to each other's findings. A third microscopist reconciled any discrepant readings (see Supplementary Information). Blood samples were processed in real time for qPCR for *P. falciparum*, however, DNA extraction and qPCR were performed at a later date and so the qPCR data did not contribute to decisions to start anti-malarial therapy.

### Parasite growth modeling

Sampling for qPCR was performed 1–2 times a day and qPCR conducted as previously described (Sheehy et al., 2012). Results were modeled using simple linear regression (Douglas et al., 2013) to estimate two key measures; liver to blood parasite inoculum (LBI) and PMR (see Supplementary Information). LBI is a quantitation of the total number of parasites released from the liver and a useful indicator of the infectious parasite load in the liver. In sporozoite CHMI studies administered by 5 infectious mosquito-bites, LBI, estimated using the geometric mean of parasitemia in the blood after the first cycle of asexual replication, ranged between 240,000 and 2,835,000 parasites, depending on CHMI center (Roestenberg et al., 2012b). PMR is the fold change in number of parasites in the blood over one lifecycle (48 h). *P. falciparum* schizonts usually contain approximately 20 merozoites (White et al., 2014). If all of these successfully invade a different red blood cell, the PMR would be 20. In CHMI studies including individuals with no NAI to malaria, PMR has been reported to range between 12 and 15 (Sheehy et al., 2012; Roestenberg et al., 2012b).

### Statistical analysis

The study was designed to assess proof of concept and group sizes were pragmatic rather than based on a formal sample size calculation for any one defined endpoint; statistical analyses were therefore primarily descriptive in nature with between group tests used sparingly and results interpreted with caution. qPCR results were compared between groups using the Mann–Whitney *U*-test (or Kruskal–Wallis test when comparing more than 2 groups). Time to treatment was presented using Kaplan–Meier survival curves and between group comparisons made using the log-rank test. Correlations were assessed using Spearman's rank correlation coefficient. Data were analyzed using GraphPad Prism version 5.03 for Windows (GraphPad Software Inc., USA) and Stata version 11 (StataCorp, Texas).

## Results

### Study participants and malaria diagnosis

**Figure 2:**
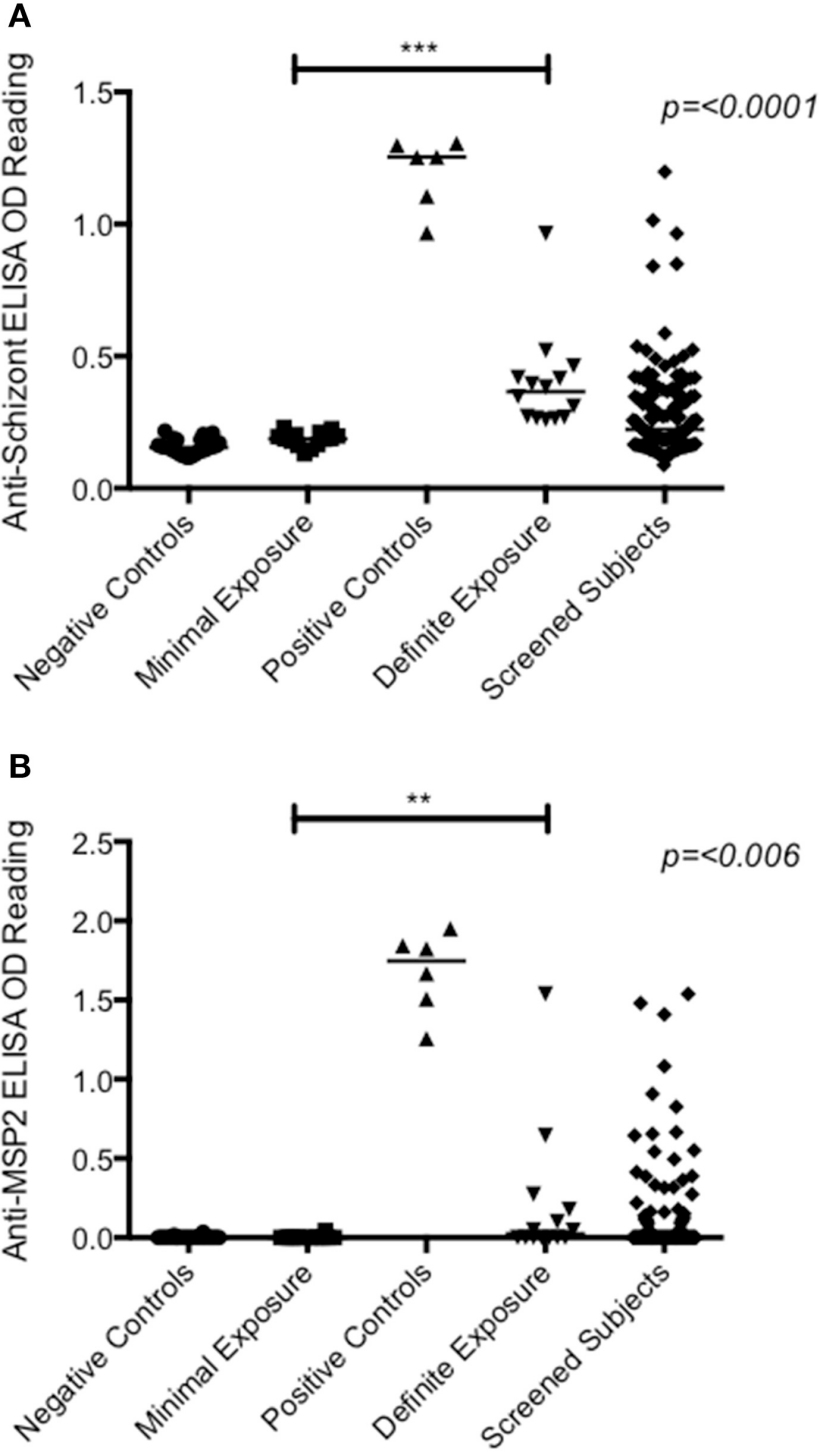
**Antibody ELISA absolute OD readings measured at screening. (A)** Anti-schizont. **(B)** Anti-MSP2. Serum diluted 1:1000. Negative controls = OD readings from UK malaria naïve adults (*n* = 30). Positive controls = OD readings from hyperimmune Kenyan adults living in malaria endemic regions (*n* = 6). Minimal exposure = subjects enrolled in groups 1, 3, and 5 (*n* = 14). Definite exposure = subjects enrolled in groups 2, 4, and 6 (*n* = 14). Screened subjects = all volunteers that had blood drawn at screening (*n* = 145). A significant difference was seen between minimally and definitely exposed volunteers for both antigens (Anti-schizont = *p* ≤ 0.0001; Anti-MSP2 = *p* = 0.006; *Mann–Whitney test*). Median values represented by lines through each dataset.

Recruitment took place in March—April 2013 when 146 volunteers were screened. Twenty-eight healthy adults (11 female and 17 male) underwent CHMI in May 2013 (Figure 1). The mean age of participants was 25 years (range 19–31 years) (Table S3). The distribution of anti-schizont and anti-MSP2 antibody OD readings of enrolled DefExp and MinExp participants at screening is shown in Figure 2. A significant correlation was seen between results from these two assays (*p* ≤ 0.0001, *R* = 0.5807). All participants received injection of PfSPZ Challenge and completed the study as scheduled.

All volunteers were diagnosed with malaria [see Table S5 for criteria for treatment and Table S6 for time to diagnosis (TTD) and parasitemia at diagnosis] with the exception of one volunteer in Group 2 (Volunteer 110), who was asymptomatic and blood film negative at all time-points, despite blood-stage infection confirmed by qPCR. This volunteer had the highest anti-schizont and MSP2 antibody OD readings at screening of all enrolled participants (Figure 2). There was no apparent relationship between dose of PfSPZ Challenge and TTD in MinExp volunteers (Median 12.6, 11.8, and 12.0 days for Groups 1, 3, and 5 respectively; *p* = 0.571 log rank test; Figure S2A) or DefExp volunteers (Median 16.4, 13.3, and 11.9 days for Groups 2, 4 and 6 respectively; *p* = 0.332 log rank test; Figure S2B); no significant difference in TTD between Group 1 and six malaria naïve volunteers who received 25,000 sporozoites IM in a previous UK study (Median TTD 12.5 vs. 13.0 days. *p* = 0.487 log rank test; Figure S2C) (Sheehy et al., 2013b) and no significant difference in TTD for MinExp and DefExp volunteers (Median 12.2 and 12.1 days respectively; *p* = 0.171 log rank test; Figure S3D).

### PfSPZ challenge reactogenicity

PfSPZ Challenge was well tolerated with only one AE deemed possibly, probably or definitely related to injection of sporozoites noted; mild injection site pain in a participant in Group 5 lasting for the day of injection only.

### PfSPZ induced clinical *Plasmodium falciparum* infection (Tables S7, S8)

**Figure 3:**
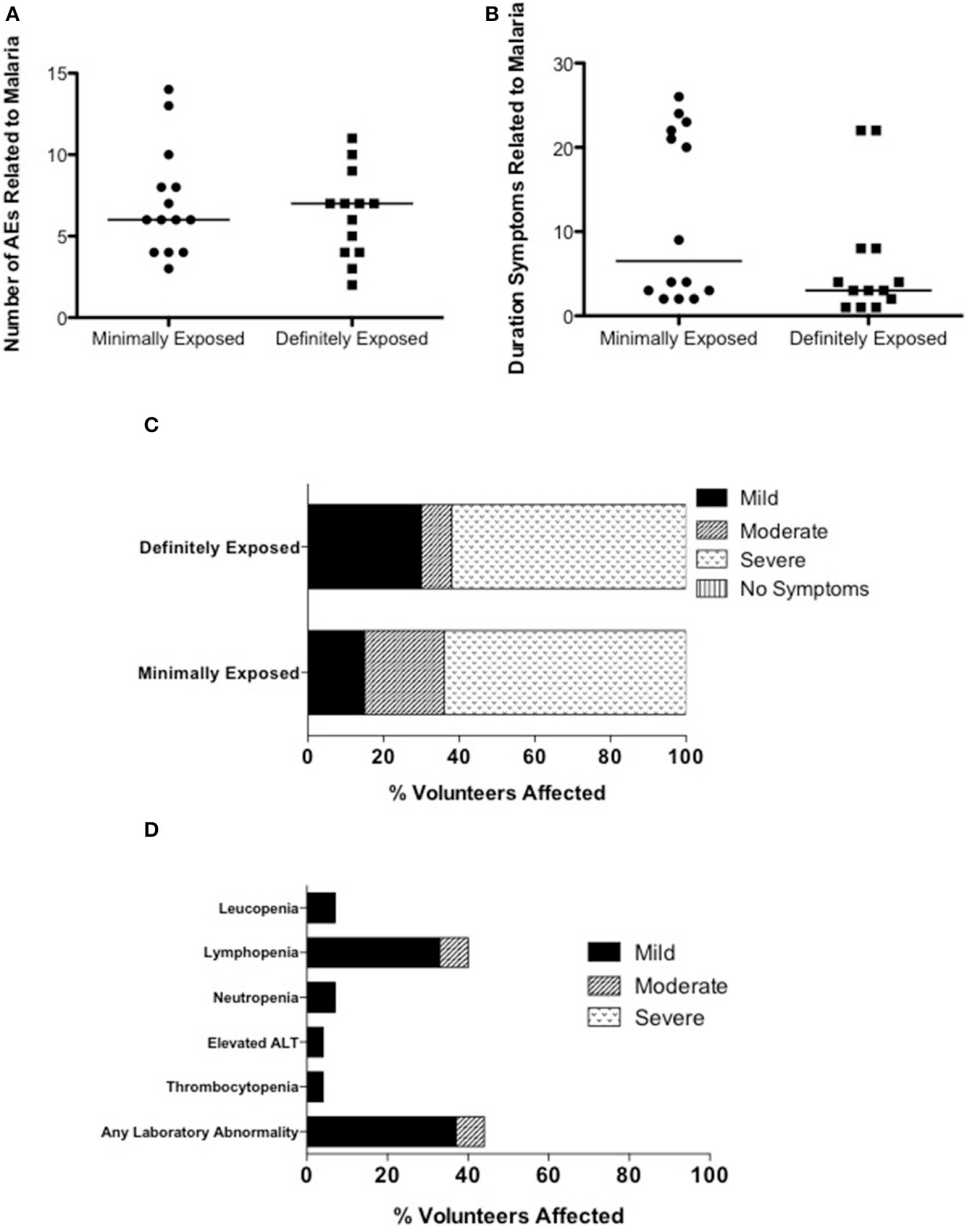
**Analysis of adverse events associated with clinical malaria. (A)** Comparison of the total number of AEs (excluding laboratory AEs) deemed possibly, probably or definitely related to clinical malaria infection in individuals diagnosed with malaria in Groups 1, 3, and 5 (Minimally exposed; mean = 7.1, median = 6.0) and Groups 2, 4, and 6 (Definitely exposed; mean = 6.3, median = 7.0) (*Mann–Whitney test*; *p* = 0.746). The median value is represented by a straight line through each plot. **(B)** Comparison of the duration of symptoms deemed possibly, probably or definitely related to clinical malaria infection in individuals diagnosed with malaria in Groups 1, 3, and 5 (Minimally exposed; mean = 11.8, median = 6.5) and Groups 2, 4, and 6 (Definitely exposed; mean = 6.3, median = 3.0) (*Mann–Whitney test*; *p* = 0.142). The median value is represented by a straight line through each plot. **(C)** Comparison of maximum severity of any symptom of clinical malaria infection between individuals diagnosed with malaria in in Groups 1, 3, and 5 (Minimally exposed) and Groups 2, 4, and 6 (Definitely exposed). **(D)** Laboratory AEs after CHMI deemed possibly, probably or definitely related to clinical *P. falciparum* infection. For “any laboratory abnormality” only the highest intensity AE per subject is counted.

No unexpected or serious AEs occurred. Twenty-two of the 27 participants diagnosed with malaria were asymptomatic at the time of diagnosis (81%; MinExp *n* = 10, DefExp *n* = 12), however all developed at least one symptom or sign consistent with malaria infection before treatment was completed (Figure 3A). The total duration of symptoms in participants with symptomatic malaria infection ranged from 1 to 26 days (median 4 days) (Figure 3B). Particularly long-lasting AEs (>20 days) occurred in 8 participants (MinExp *n* = 6, DefExp *n* = 2) and included headache (*n* = 5), anorexia (*n* = 4), low back pain (*n* = 2), fatigue (*n* = 2), and arthralgia (*n* = 1). There was no difference in the number, duration, or severity of symptoms of malaria between MinExp and DefExp participants (Figures 3A–C). Seventeen out of 27 participants diagnosed with *P. falciparum* (63%) experienced at least one AE post CHMI that was severe in intensity (Figure 3C). Three volunteers diagnosed with malaria (11%) had a fever (>37.5°C) prior to diagnosis (MinExp *n* = 2, DefExp *n* = 1) and 17 participants (63%) developed a fever following treatment (MinExp *n* = 8, DefExp *n* = 9). Safety bloods taken 9 days after initiation of CHMI (dC+9), dC+35, dC+90 and within 24 h of diagnosis demonstrated transient laboratory abnormalities at frequencies and severities expected following *P. falciparum* infection in CHMI studies (Figure 3D) (Epstein et al., 2007; Sheehy et al., 2012).

### Parasitemia measured by qPCR

**Figure 4:**
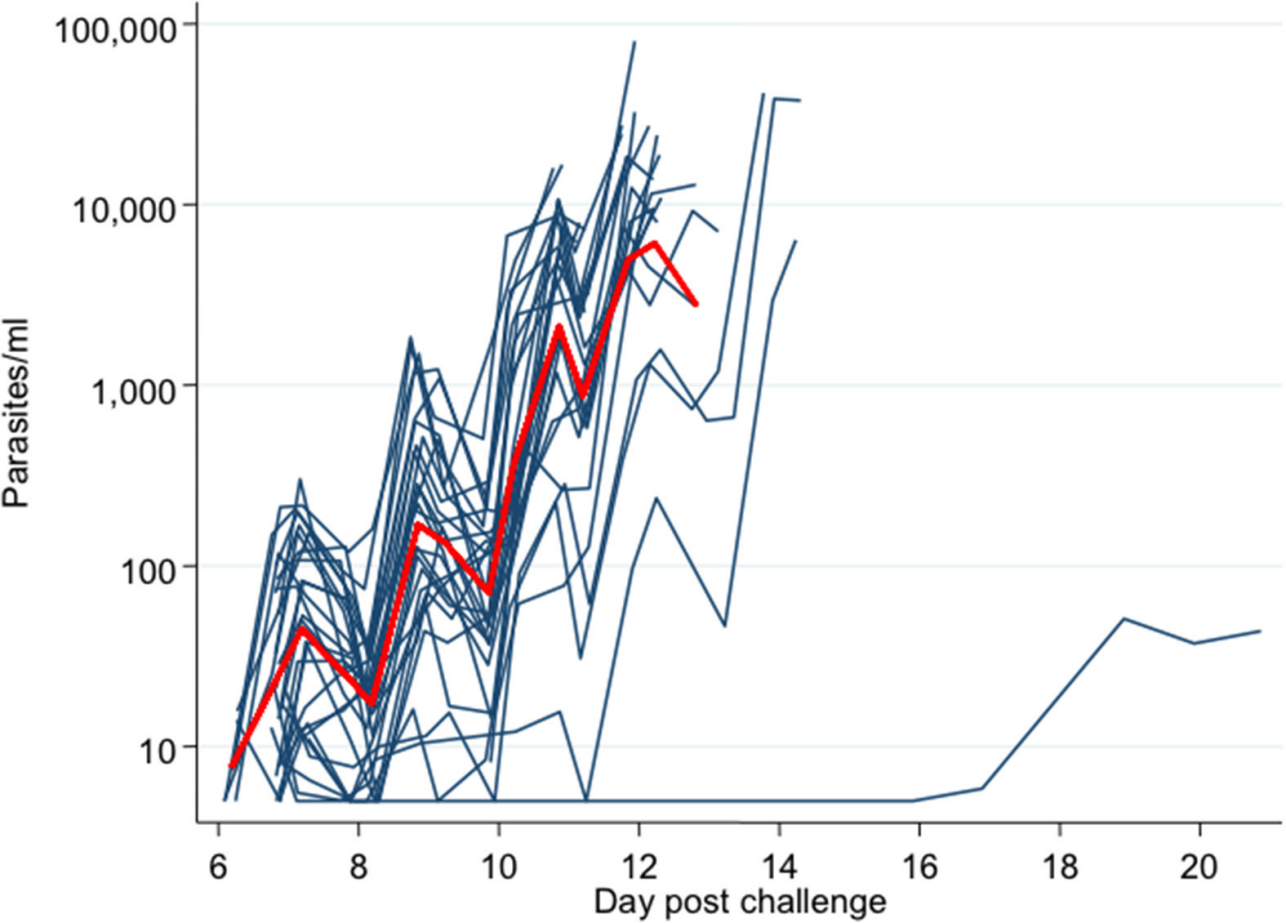
**qPCR-measured parasite density for each individual subject**. Y-axis = parasites/mL. X-axis = days post challenge (calculated from hours post challenge). Red line = mean parasite density over time for all subjects.

**Figure 5:**
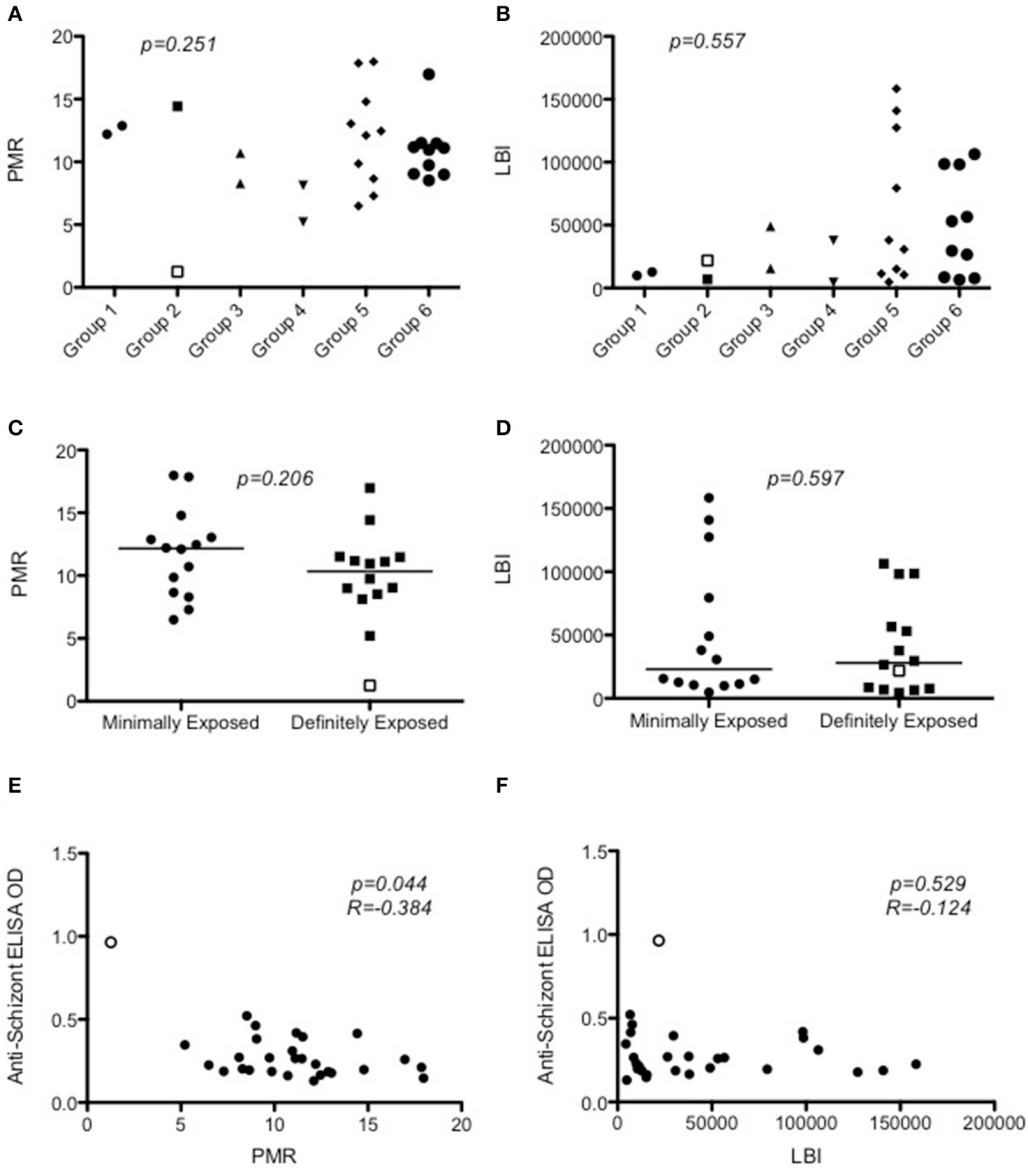
**Modeling of qPCR-measured parasite density to calculate parasite multiplication rates and liver-to-blood inocula**. **(A)** Dot plot of PMR according to Group (*p* = 0.251; *Kruskal–Wallis test*). **(B)** Dot plot of LBI according to Group (*p* = 0.547; *Kruskal–Wallis test*). **(C)** PMR according to prior exposure to malaria (*p* = 0.206; *Mann–Whitney test*). The median value is represented by a straight line through each plot **(D)** LBI according to prior exposure to malaria (*p* = 0.700; *Mann–Whitney test*). Median value is represented by a straight line through each plot. **(E)** Relationship between PMR and anti-schizont ELISA OD measured at screening (*Spearman rank* = −0.384; *p* = 0.044). **(F)** Relationship between LBI and anti-schizont ELISA OD measured at screening (*Spearman rank* = −0.123; *p* = 0.534). In all graphs Volunteer 110′s data points are represented as open points. PMR = parasite multiplication rate (fold change in parasites/mL over 48 h). LBI = Liver-to-blood Inocula (total number of parasites released from liver).

**Figure 6:**
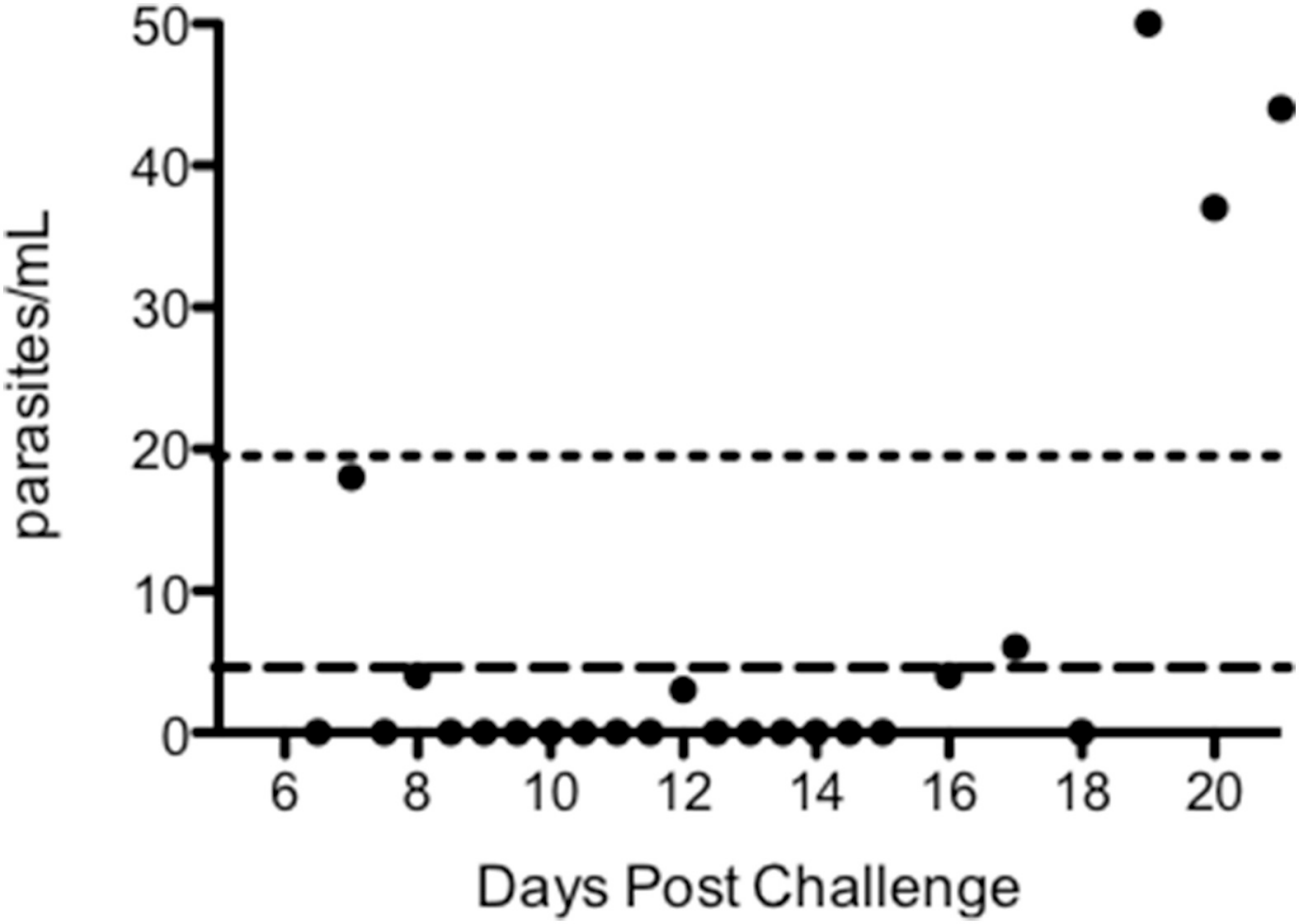
**qPCR results post-challenge for volunteer 110, group 2**. Long dashed line = lower limit of detection (i.e., a probability of >50% of ≥1 positive result among three replicate PCR reactions) for qPCR assay (5 parasites/mL). Short dashed line = lower limit of quantification (defined as %CV < 20%) for qPCR assay (20 parasites/mL).

Figure 4 and Figure S3 plot the qPCR results over time for each individual in the trial. There was no significant difference in PMR or LBI between groups (*p* = 0.251 and *p* = 0.557 respectively; *Kruskall–Wallis test* Figures 5A,B) or between MinExp and DefExp participants (*p* = 0.206 and *p* = 0.597 respectively; *Mann–Whitney test* Figures 5C,D). A significant correlation was seen between anti-schizont antibody OD at screening and PMR (*Spearman rank =* -pearman *p* = 0.044) but not LBI (*Spearman rank* = −0.124, *p* = 0.529) (Figures 5E,F). No significant correlation was seen between anti-schizont OD and PMR when volunteer 110 was excluded from the analysis (*p* = 0.112, *R* = 0.313). In contrast to previous published data, no relationship was seen between LBI and PMR (*Spearman rank* = −0.176, *p* = 0.371) (Douglas et al., 2013). The qPCR data for Volunteer 110 are shown in Figure 6. Volunteer 110 had a notably reduced PMR (1.3) in comparison to the other 27 volunteers (median 11.1). Importantly, Volunteer 110 was qPCR positive on dC+7 (mean 18 p/mL; triplicate readings; 14, 38, and 0 p/mL, positivity at this time-point confirmed using a second PCR method, Murphy et al., 2012) and then subsequently qPCR negative until dC+19, suggesting control of blood-stage parasite growth rather than an extremely low or undetectable LBI. Moreover, the LBI in this volunteer was mid-range, in comparison to all other challenges (Figures 5B,D).

We compared MSP2 genotypes of the blood-stage infections using the Agilent Bioanalyzer to examine the qPCR products following amplification of the MSP2 gene (Liljander et al., 2009). The fragment sizes were similar to within 10 base pairs (within the error of the method) and therefore all infections were clonal (NF54) and assumed to result from injection with PfSPZ Challenge rather than being naturally acquired.

## Discussion

The primary aim of our pilot study was to establish the parameters for safely and practically performing CHMI studies in Kenya. Given the unknown effects of prior exposure to malaria on CHMI, we sought to include individuals with both MinExp and DefExp to *P. falciparum* and therefore, presumed varying levels of NAI. Our study has shown that the CHMI model using PfSPZ Challenge is safe in African adults with no significant differences in AEs seen between DefExp and MinExp volunteers. Importantly, we observed a similar safety profile to that reported in malaria-naïve subjects who underwent CHMI, with the exception that Kenyan participants experienced AEs of a notably longer duration than malaria-naïve volunteers, the reason for which is unclear (Roestenberg et al., 2012b; Sheehy et al., 2012, 2013b).

Despite screening nearly 150 volunteers, only a third were eligible to participate in the study (Figure S1). As the first CHMI study undertaken in Kenya, the exclusion criteria were extensive. Issues likely to be pertinent to other trials in Africa, such as the high degree of previously undiagnosed co-morbidities, asymptomatic qPCR positivity and hemoglobinopathies, restricted recruitment. For future Kenyan CHMI studies we anticipate screening large numbers of volunteers to identify a sufficient number of healthy, eligible adults.

Of our screened cohort, only 5 individuals had anti-schizont OD readings of the same order as “hyperimmune” controls (Figure 2). Due to a high degree of previously undiagnosed co-morbidities in these 5 individuals, only one of these volunteers was subsequently eligible to participate in the study. In practice therefore, our study included subjects with varying but probably low to moderate prior exposure to *P. falciparum*. Nevertheless, there was no overlap between the anti-schizont reactivities of those with definite vs. minimal exposure. If future Kenyan CHMI studies seek to enroll “hyperimmune” adults, it will be important to either screen a considerably larger sample of individuals or to do targeted screening.

However, despite the lack of “hyperimmune” individuals in our study, we still have preliminary evidence of the potential of the CHMI model to detect differences in individuals' NAI to *P. falciparum*. This is demonstrated first by the relationship between the anti-schizont OD reading at screening and PMR post-CHMI (Figure 5E), and second by the case of the individual with the highest anti-schizont ELISA OD at screening (Volunteer 110) who remained asymptomatic and blood-film negative despite qPCR confirmed *P. falciparum* infection. Comparison of PMRs from our study with the only other published measurement of PMR in the context of NAI (Douglas et al., 2011) showed that this volunteer had a similar PMR to those measured in Gambian adults participating as controls in a malaria vaccine field efficacy study (Imoukhuede et al., 2007; Douglas et al., 2011). However, it should be noted these latter adult volunteers had presumably been infected multiple times before with local circulating strains against which they had developed effective immunity, in contrast to our study which used the historical laboratory reference strain NF54, which, although of African origin, may not completely reflect the strains against which our volunteers were naturally exposed in the past. Other volunteers in our study appeared to have PMRs broadly similar to malaria naïve controls post CHMI administered by mosquito bite (Roestenberg et al., 2012b; Sheehy et al., 2012; Douglas et al., 2013). Nevertheless, key methodological differences in qPCR analysis and study design (including “drug clearance” before qPCR sampling) between these studies and limited other trials reporting vaccine-induced reduction in parasite growth post-CHMI (Thompson et al., 2008; Spring et al., 2009), make comparison of PMRs across these various settings largely difficult to interpret (Sheehy et al., 2013a).

Examination of Volunteer 110's qPCR data over time show a positive qPCR result on dC+7.0 followed by a run of mostly negative results, until dC+19.0 when all subsequent qPCR results became positive. Whilst it is not possible to draw definite conclusions regarding the mechanism by which this volunteer controlled parasite growth, this pattern of qPCR positivity could suggest the first wave of merozoites released from the liver invaded red blood cells and thus were detectable at dC+7.0 post-CHMI and not completely controlled by anti-merozoite immunity. The subsequent run of negative qPCR results would be consistent with control of parasite growth mediated by existing anti-PfEMP1 or anti-infected red blood cell (iRBC) antibodies, followed by loss of control of parasite growth. This could occur following a *var* gene switch, leading to a change in variant surface antigen to one that is not recognized by existing antibodies (Peters et al., 2002; Warimwe et al., 2009). Further work will now assess assays of anti-merozoite immunity and *var* gene expression during the blood-stage of infection in Volunteer 110 to evaluate this hypothesis. Alternatively, there is a possibility that the late increase in qPCR seen in this volunteer represented gametocytaemia. Given that no asexual parasites were seen at any time-point on blood-film we think this unlikely, however future trials could consider performing gametocyte-specific qPCR in such scenarios to test for this possibility (Wampfler et al., 2013).

Our study importantly confirms the infectivity of PfSPZ Challenge in malaria exposed individuals, supporting the future use of PfSPZ Challenge in malaria endemic regions to test vaccines and anti-malaria drugs. In contrast to previous clinical data (Sheehy et al., 2013b), our study showed no significant increase in infectivity (measured by LBI, TTD, or proportion of participants infected) with increasing dose of PfSPZ Challenge IM, suggesting 25,000 sporozoites may be a sufficient dose to ensure infection when administered IM. Recent trials using PfSPZ Challenge administered intravenously (IV) suggest this route of administration can reliably ensure successful infection in CHMI trials with lower doses of sporozoites than those required when administered by other routes (Hoffman, Pers. Commun.). Since IV administration can reduce the variation in LBI between volunteers and allow an infectious dose more closely approximating that seen in the field, this route of injection of PfSPZ Challenge may be the way forward for future CHMI studies aiming to examine the dynamics of NAI.

### Conflict of interest statement

Sanaria Inc. manufactured PfSPZ Challenge. Thus, all authors associated with Sanaria (Peter F. Billingsley, Eric R. James, Anusha Gunasekera, B. Kim L. Sim, Adam Richman, Yonas Abebe, Stephen L. Hoffman) have potential conflicts of interest.

## Supplementary material

Figure S1 **Primary reasons for exclusion of volunteers**. Of note, some individuals met multiple exclusion criteria. This figure illustrates the primary reason for exclusion for each volunteer. HIV, human immunodeficiency virus; ECG, electrocardiogram; PCR, quantitative polymerase chain reaction for P. falciparum; ALT, Alanine transaminase; Homo α thal, Homozygous α thalassemia; Hetero α thal, Heterozygous α thalassemia; β HCG, β Human Chorionic Gonadotropin.

Figure S2 **Kaplan–Meier analysis of Time to Diagnosis**. Time to diagnosis (TTD) calculated in hours and converted to days from injection of PfSPZ Challenge and meeting end-point criteria. **(A)** Effect of dose of PfSPZ Challenge on TTD in minimally exposed participants. Median pre-patent period = 12.6 days for Group 1, 11.8 days for Group 3 and 12.0 days for Group 5 (*Log-rank test*; *p* = 0.571). **(B)** Effect of dose of PfSPZ Challenge on TTD in Definitely exposed participants. Median pre-patent period = 16.4 days for Group 2, 13.3 days for Group 4 and 11.9 days for Group 6. **(C)** Comparison of pre-patent period between malaria naïve UK volunteers (*n* = 6) (Sheehy et al., 2013b) and Kenyan minimally exposed volunteers (Group 1; *n* = 2) receiving PfSPZ Challenge 25,000 IM. Median pre-patent period = 12.9 days for UK volunteers and 12.6 days for Group 1 (*Log-rank test*; *p* = 0.487). **(D)** Effect of prior exposure to *P. falciparum* on TTD. Minimally exposed = Groups 1, 3, and 5. Definitely exposed = Groups 2, 4, and 6. Median pre-patient period = 12.2 days for Minimally exposed and 12.1 days for Definitely exposed (*Log-rank test*; *p* = 0.171).

Figure S3 **qPCR-measured parasite density over time for each individual subject**. Y-axis = Parasites/mL measured by qPCR. X-axis = days post-injection of PfSPZ Challenge. Black subtitles indicate participant identification numbers.

## References

[B1] Allison A. C. (1954). Protection afforded by sickle-cell trait against subtertian malareal infection. Br. Med. J. 1, 290–294. 10.1136/bmj.1.4857.290 13115700 PMC2093356

[B2] Bray R. S. Gunders A. E. Burgess R. W. Freeman J. B. Etzel E. Guttuso C.. (1962). The inoculation of semi-immune Africans with sporozoites of Laverania falcipara (*Plasmodium falciparum*) in Liberia. Riv. Malariol. 41, 199–210. 14015116

[B3] Bruce-Chwatt L. J. (1963a). A longitudinal longitudinal survey of natural malaria infection in a group of West African adults. I. West Afr. Med. J. 12, 141–173. 14052649

[B4] Bruce-Chwatt L. J. (1963b). A longitudinal survey of natural malaria infection in a group of West African adults. West Afr. Med. J. 12, 199–217. 14056767

[B5] Chulay J. D. Schneider I. Cosgriff T. M. Hoffman S. L. Ballou W. R. Quakyi I. A.. (1986). Malaria transmitted to humans by mosquitoes infected from cultured *Plasmodium falciparum*. Am. J. Trop. Med. Hyg. 35, 66–68. 3511753 10.4269/ajtmh.1986.35.66

[B6] Douglas A. D. Andrews L. Draper S. J. Bojang K. Milligan P. Gilbert S. C.. (2011). Substantially reduced pre-patent parasite multiplication rates are associated with naturally acquired immunity to *Plasmodium falciparum*. J. Infect. Dis. 203, 1337–1340. 10.1093/infdis/jir033 21459819 PMC3398130

[B7] Douglas A. D. Edwards N. J. Duncan C. J. Thompson F. M. Sheehy S. H. O'hara G. A.. (2013). Comparison of modeling methods to determine liver-to-blood Inocula and parasite multiplication rates during controlled human malaria infection. J. Infect. Dis. 208, 340–345. 10.1093/infdis/jit156 23570846 PMC3685228

[B8] Drakeley C. J. Corran P. H. Coleman P. G. Tongren J. E. Mcdonald S. L. Carneiro I.. (2005). Estimating medium- and long-term trends in malaria transmission by using serological markers of malaria exposure. Proc. Natl. Acad. Sci. U.S.A. 102, 5108–5113. 10.1073/pnas.0408725102 15792998 PMC555970

[B9] Duncan C. J. Hill A. V. Ellis R. D. (2012). Can growth inhibition assays (GIA) predict blood-stage malaria vaccine efficacy? Hum. Vaccin. Immunother. 8, 706–714. 10.4161/hv.19712 22508415 PMC3495712

[B10] Epstein J. E. Rao S. Williams F. Freilich D. Luke T. Sedegah M.. (2007). Safety and clinical outcome of experimental challenge of human volunteers with *Plasmodium falciparum*-infected mosquitoes: an update. J. Infect. Dis. 196, 145–154. 10.1086/518510 17538895

[B11] Hill D. L. Eriksson E. M. Carmagnac A. B. Wilson D. W. Cowman A. F. Hansen D. S.. (2012). Efficient measurement of opsonising antibodies to *Plasmodium falciparum* merozoites. PLoS ONE 7:e51692. 10.1371/journal.pone.0051692 23300556 PMC3530572

[B12] Imoukhuede E. B. Andrews L. Milligan P. Berthoud T. Bojang K. Nwakanma D.. (2007). Low-level malaria infections detected by a sensitive polymerase chain reaction assay and use of this technique in the evaluation of malaria vaccines in an endemic area. Am. J. Trop. Med. Hyg. 76, 486–493. 17360872 PMC3836239

[B13] Joos C. Marrama L. Polson H. E. Corre S. Diatta A. M. Diouf B.. (2010). Clinical protection from falciparum malaria correlates with neutrophil respiratory bursts induced by merozoites opsonized with human serum antibodies. PLoS ONE 5:e9871. 10.1371/journal.pone.0009871 20360847 PMC2845614

[B14] Langhorne J. Ndungu F. M. Sponaas A. M. Marsh K. (2008). Immunity to malaria: more questions than answers. Nat. Immunol. 9, 725–732. 10.1038/ni.f.205 18563083

[B15] Liljander A. Wiklund L. Falk N. Kweku M. Martensson A. Felger I.. (2009). Optimization and validation of multi-coloured capillary electrophoresis for genotyping of *Plasmodium falciparum* merozoite surface proteins (msp1 and 2). Malar. J. 8:78. 10.1186/1475-2875-8-78 19386138 PMC2680902

[B16] Marsh K. Otoo L. Hayes R. J. Carson D. C. Greenwood B. M. (1989). Antibodies to blood stage antigens of *Plasmodium falciparum* in rural Gambians and their relation to protection against infection. Trans. R. Soc. Trop. Med. Hyg. 83, 293–303. 10.1016/0035-9203(89)90478-1 2694458

[B17] Mccallum F. J. Persson K. E. Mugyenyi C. K. Fowkes F. J. Simpson J. A. Richards J. S.. (2008). Acquisition of growth-inhibitory antibodies against blood-stage *Plasmodium falciparum*. PLoS ONE 3:e3571. 10.1371/journal.pone.0003571 18958278 PMC2570221

[B18] Mccarthy J. S. Sekuloski S. Griffin P. M. Elliott S. Douglas N. Peatey C.. (2011). A pilot randomised trial of induced blood-stage *Plasmodium falciparum* infections in healthy volunteers for testing efficacy of new antimalarial drugs. PLoS ONE 6:e21914. 10.1371/journal.pone.0021914 21887214 PMC3159571

[B19] Murphy S. C. Prentice J. L. Williamson K. Wallis C. K. Fang F. C. Fried M.. (2012). Real-time quantitative reverse transcription PCR for monitoring of blood-stage *Plasmodium falciparum* infections in malaria human challenge trials. Am. J. Trop. Med. Hyg. 86, 383–394. 10.4269/ajtmh.2012.10-0658 22403305 PMC3284350

[B20] Nieman A. E. De Mast Q. Roestenberg M. Wiersma J. Pop G. Stalenhoef A.. (2009). Cardiac complication after experimental human malaria infection: a case report. Malar. J. 8:277. 10.1186/1475-2875-8-277 19958549 PMC2794284

[B21] Osier F. H. Fegan G. Polley S. D. Murungi L. Verra F. Tetteh K. K.. (2008). Breadth and magnitude of antibody responses to multiple *Plasmodium falciparum* merozoite antigens are associated with protection from clinical malaria. Infect. Immun. 76, 2240–2248. 10.1128/IAI.01585-07 18316390 PMC2346713

[B22] Peters J. Fowler E. Gatton M. Chen N. Saul A. Cheng Q. (2002). High diversity and rapid changeover of expressed var genes during the acute phase of *Plasmodium falciparum* infections in human volunteers. Proc. Natl. Acad. Sci. U.S.A. 99, 10689–10694. 10.1073/pnas.162349899 12142467 PMC125014

[B23] Polley S. D. Conway D. J. Cavanagh D. R. Mcbride J. S. Lowe B. S. Williams T. N.. (2006). High levels of serum antibodies to merozoite surface protein 2 of *Plasmodium falciparum* are associated with reduced risk of clinical malaria in coastal Kenya. Vaccine 24, 4233–4246. 10.1016/j.vaccine.2005.06.030 16111789

[B24] Roestenberg M. Bijker E. M. Sim B. K. Billingsley P. F. James E. R. Bastiaens G. J.. (2013). Controlled human malaria infections by intradermal injection of cryopreserved *Plasmodium falciparum* sporozoites. Am. J. Trop. Med. Hyg. 88, 5–13. 10.4269/ajtmh.2012.12-0613 23149582 PMC3541746

[B25] Roestenberg M. De Vlas S. J. Nieman A. E. Sauerwein R. W. Hermsen C. C. (2012a). Efficacy of preerythrocytic and blood-stage malaria vaccines can be assessed in small sporozoite challenge trials in human volunteers. J. Infect. Dis. 206, 319–323. 10.1093/infdis/jis355 22615322

[B26] Roestenberg M. O'hara G. A. Duncan C. J. Epstein J. E. Edwards N. J. Scholzen A.. (2012b). Comparison of clinical and parasitological data from controlled human malaria infection trials. PLoS ONE 7:e38434. 10.1371/journal.pone.0038434 22701640 PMC3372522

[B27] Sauerwein R. W. Roestenberg M. Moorthy V. S. (2011). Experimental human challenge infections can accelerate clinical malaria vaccine development. Nat. Rev. Immunol. 11, 57–64. 10.1038/nri2902 21179119

[B28] Sheehy S. H. Douglas A. D. Draper S. J. (2013a). Challenges of assessing the clinical efficacy of asexual blood-stage *Plasmodium falciparum* malaria vaccines. Hum. Vaccin. Immunother. 9, 1831–1840. 10.4161/hv.25383 23778312 PMC3906345

[B29] Sheehy S. H. Duncan C. J. Elias S. C. Choudhary P. Biswas S. Halstead F. D.. (2012). ChAd63-MVA-vectored blood-stage malaria vaccines targeting MSP1 and AMA1: assessment of efficacy against mosquito bite challenge in humans. Mol. Ther. 20, 2355–2368. 10.1038/mt.2012.223 23089736 PMC3519995

[B30] Sheehy S. H. Spencer A. J. Douglas A. D. Sim B. K. Longley R. J. Edwards N. J.. (2013b). Optimising controlled human malaria infection studies using cryopreserved parasites administered by needle and syringe. PLoS ONE 8:e65960. 10.1371/journal.pone.0065960 23823332 PMC3688861

[B31] Shekalaghe S. Rutaihwa M. Billingsley P. F. Chemba M. Daubenberger C. A. James E.. (2014). Controlled human malaria infection of tanzanians by intradermal injection of aseptic, purified, cryopreserved *Plasmodium falciparum* sporozoites. Am. J. Trop. Med. Hyg. 91, 471–480. 10.4269/ajtmh.14-0119 25070995 PMC4155546

[B32] Spring M. D. Cummings J. F. Ockenhouse C. F. Dutta S. Reidler R. Angov E.. (2009). Phase 1/2a study of the malaria vaccine candidate apical membrane antigen-1 (AMA-1) administered in adjuvant system AS01B or AS02A. PLoS ONE 4:e5254. 10.1371/journal.pone.0005254 19390585 PMC2669163

[B33] Thompson F. M. Porter D. W. Okitsu S. L. Westerfeld N. Vogel D. Todryk S.. (2008). Evidence of blood stage efficacy with a virosomal malaria vaccine in a phase IIa clinical trial. PLoS ONE 3:e1493. 10.1371/journal.pone.0001493 18231580 PMC2204057

[B34] Tippett E. Fernandes L. A. Rogerson S. J. Jaworowski A. (2007). A novel flow cytometric phagocytosis assay of malaria-infected erythrocytes. J. Immunol. Methods 325, 42–50. 10.1016/j.jim.2007.05.012 17658546

[B35] Van Meer M. P. Bastiaens G. J. Boulaksil M. De Mast Q. Gunasekera A. Hoffman S. L.. (2014). Idiopathic acute myocarditis during treatment for controlled human malaria infection: a case report. Malar. J. 13:38. 10.1186/1475-2875-13-38 24479524 PMC3909449

[B36] Wampfler R. Mwingira F. Javati S. Robinson L. Betuela I. Siba P.. (2013). Strategies for detection of Plasmodium species gametocytes. PLoS ONE 8:e76316. 10.1371/journal.pone.0076316 24312682 PMC3848260

[B37] Warimwe G. M. Keane T. M. Fegan G. Musyoki J. N. Newton C. R. Pain A.. (2009). *Plasmodium falciparum* var gene expression is modified by host immunity. Proc. Natl. Acad. Sci. U.S.A. 106, 21801–21806. 10.1073/pnas.0907590106 20018734 PMC2792160

[B38] White N. J. Pukrittayakamee S. Hien T. T. Faiz M. A. Mokuolu O. A. Dondorp A. M. (2014). Malaria. Lancet 383, 723–735. 10.1016/S0140-6736(13)60024-0 23953767

