# Supplementary figures and images for: Evaluating controlled human malaria infection in Kenyan adults with varying degrees of prior exposure to Plasmodium falciparum using sporozoites administered by intramuscular injection
Source: Front Microbiol. 2014 Dec 12;5:686. doi: 10.3389/fmicb.2014.00686 (PMC4264479; doi:10.3389/fmicb.2014.00686)

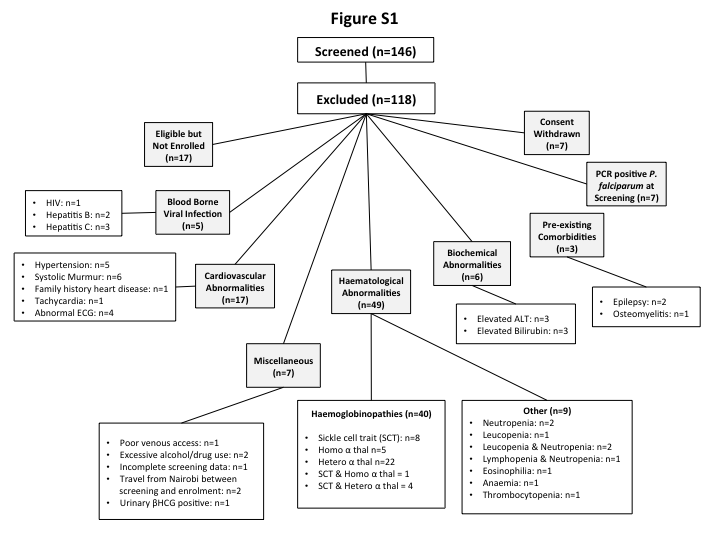

Supplement: Figure S1 — Primary reasons for exclusion of volunteers. Of note, some individuals met multiple exclusion criteria. This figure illustrates the primary reason for exclusion for each volunteer. HIV, human immunodeficiency virus; ECG, electrocardiogram; PCR, quantitative polymerase chain reaction for P. falciparum; ALT, Alanine transaminase; Homo α thal, Homozygous α thalassemia; Hetero α thal, Heterozygous α thalassemia; β HCG, β Human Chorionic Gonadotropin. [file Image1.TIFF]

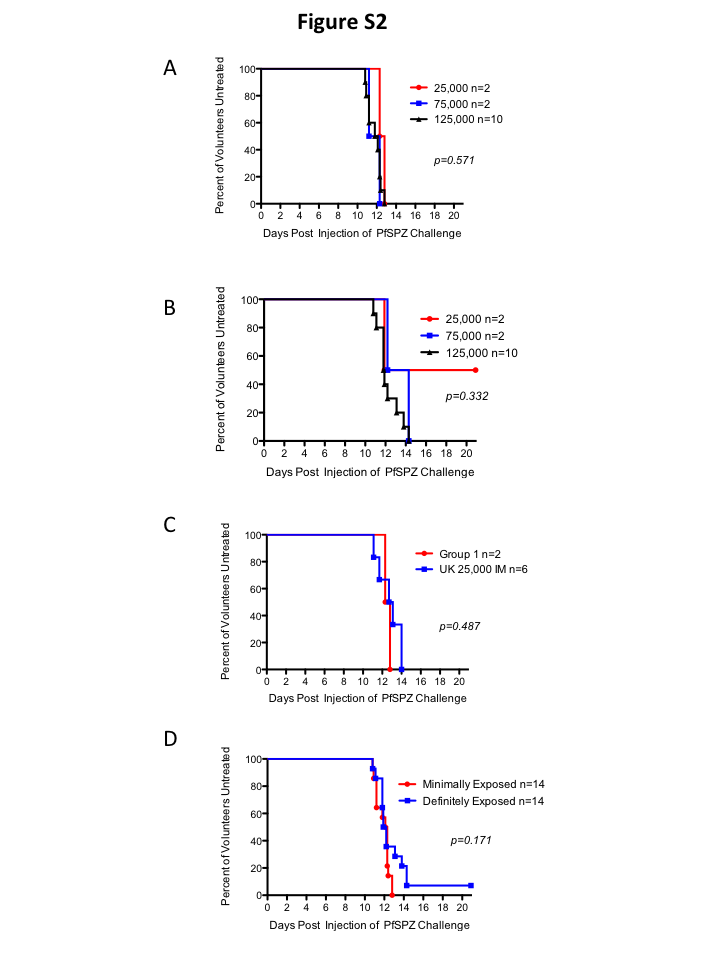

Supplement: Figure S2 — Kaplan–Meier analysis of Time to Diagnosis. Time to diagnosis (TTD) calculated in hours and converted to days from injection of PfSPZ Challenge and meeting end-point criteria. (A) Effect of dose of PfSPZ Challenge on TTD in minimally exposed participants. Median pre-patent period = 12.6 days for Group 1, 11.8 days for Group 3 and 12.0 days for Group 5 (Log-rank test; p = 0.571). (B) Effect of dose of PfSPZ Challenge on TTD in Definitely exposed participants. Median pre-patent period = 16.4 days for Group 2, 13.3 days for Group 4 and 11.9 days for Group 6. (C) Comparison of pre-patent period between malaria naïve UK volunteers (n = 6) (Sheehy et al., 2013b) and Kenyan minimally exposed volunteers (Group 1; n = 2) receiving PfSPZ Challenge 25,000 IM. Median pre-patent period = 12.9 days for UK volunteers and 12.6 days for Group 1 (Log-rank test; p = 0.487). (D) Effect of prior exposure to P. falciparum on TTD. Minimally exposed = Groups 1, 3, and 5. Definitely exposed = Groups 2, 4, and 6. Median pre-patient period = 12.2 days for Minimally exposed and 12.1 days for Definitely exposed (Log-rank test; p = 0.171). [file Image2.TIFF]

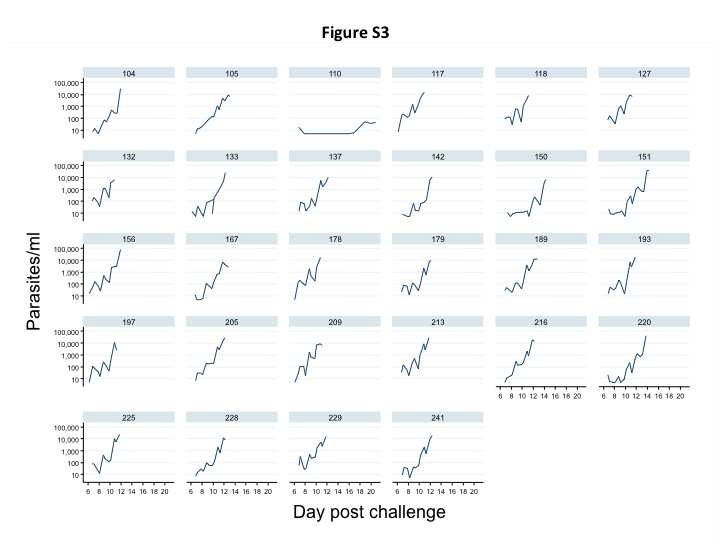

Supplement: Figure S3 — qPCR-measured parasite density over time for each individual subject. Y-axis = Parasites/mL measured by qPCR. X-axis = days post-injection of PfSPZ Challenge. Black subtitles indicate participant identification numbers. [file Image3.TIFF]
