# Supplementary material for: Evaluating controlled human malaria infection in Kenyan adults with varying degrees of prior exposure to Plasmodium falciparum using sporozoites administered by intramuscular injection
Source: Front Microbiol. 2014 Dec 12;5:686. doi: 10.3389/fmicb.2014.00686 (PMC4264479; doi:10.3389/fmicb.2014.00686)
Supplement: Supplementary file 4 [file DataSheet1.DOCX]

**SUPPLEMENTARY INFORMATION**

**Inclusion Criteria:**

- Healthy adults aged 18 to 40 years.
- Minimum of 4 completed years of secondary education.
- Able and willing, in the Investigator’s opinion, to comply with all study requirements.
- Informed consent to undergo CHMI.
- Answer all questions on the informed consent questionnaire correctly.
- Willingness to take a course of curative anti-malaria medication.
- Agreement to stay in an in-patient unit during a part of the study (from day of administration of PfSPZ Challenge until completion of curative course of anti-malarial therapy given either at malaria diagnosis or day 21 post-administration of PfSPZ Challenge).
- Use of effective method of contraception for duration of study (women only).

**Exclusion criteria**

Any of the following constituted an exclusion criterion:

- PCR positive for *P. falciparum* parasites at screening.
- Use of systemic antibiotics with known antimalarial activity within 30 days of administration of PfSPZ Challenge (e.g. trimethoprim-sulfamethoxazole, doxycycline, tetracycline, clindamycin, erythromycin, fluoroquinolones and azithromycin).
- Receipt of an investigational product in the 30 days preceding enrolment, or planned receipt during the study period.
- Current participation in another clinical trial or recent participation within 12 weeks of enrolment.
- Prior receipt of an investigational malaria vaccine.
- Any confirmed or suspected immunosuppressive or immunodeficient state, including HIV infection; asplenia; recurrent, severe infections and chronic (more than 14 days) immunosuppressant medication within the past 6 months (inhaled and topical steroids are allowed).
- Use of immunoglobulins or blood products within 3 months prior to enrolment.
- Sickle cell trait or heterozygous or homozygous alpha thalassemia.
- A history of allergic disease or reactions likely to be exacerbated by malaria infection.
- Contraindications to atovaquone/proguanil hydrochloride.
- History of cancer (except basal cell carcinoma of the skin and cervical carcinoma *in situ*).
- History of serious psychiatric condition that may affect participation in the study.
- Any other serious chronic illness requiring hospital specialist supervision.
- Women only; pregnancy, intention to become pregnant or breast-feeding during study.
- Suspected or known current alcohol abuse.
- Suspected or known injecting drug abuse.
- Seropositive for hepatitis B surface antigen (HBsAg).
- Seropositive for hepatitis C virus (antibodies to HCV) with PCR positive for Hepatitis C.
- Positive family history in 1st and 2nd degree relatives < 50 years old for cardiac disease.
- Any clinically significant abnormal finding on biochemistry or haematology blood tests, urinalysis or clinical examination.
- Any other significant disease, disorder or finding which may significantly increase the risk to the volunteer because of participation in the study, affect the ability of the volunteer to participate in the study or impair interpretation of the study data.

***Exclusion Criterion on Day of Challenge***

- Acute disease, defined as moderate or severe illness with or without fever (temperature >37.5^o^C).

**Hemoglobinopathy Screening**

Genotyping for sickle haemoglobin (HbS) and the common African 3.7-kb α-globin α^+^-thalassaemia deletion were conducted by PCR as described in detail previously.([Chong et al., 2000](#_ENREF_1); [Waterfall and Cobb, 2001](#_ENREF_6); [Williams et al., 2005](#_ENREF_7)) Participants with one α-globin deletion (-α/αα) were defined as heterozygous α-thalassemia, and those with two α-globin deletions (-α/-α) were defined as homozygous α-thalassemia. Volunteers were excluded if they were heterozygous or homozygous for α-thalassemia or sickle haemoglobin.

**Assessing Anti-Schizont and Anti-Merozoite Antibody Titres at Screening**

Briefly, individual wells of Dynex Immunolon 4HBX ELISA plates (Dynex Technologies Inc) were coated with 50ng of recombinant MSP2 (Dd2 allele)([Taylor et al., 1995](#_ENREF_5)) per 100µL of carbonate coating buffer (15mM Na_2_CO_3_, 35mM NaHCO_3_, pH 9.3). *P. falciparum* schizont extract (A4 and 3D7 parasite strains) was coated onto wells in PBS.([Ndungu et al., 2002](#_ENREF_4)) Plates were incubated overnight at 4^o^C, before washing four times in PBS/Tween (Phosphate Buffered Saline/0.05% Tween 20), and blocking for 5 hours at room temperature with 1% skimmed milk in PBS/Tween (blocking buffer). Following this, wells were washed again and incubated overnight at 4^o^C with 100μL of test sera (1/1000 dilution in blocking buffer). Plates were then washed four times and incubated for 3 hours at room temperature with 100μL of HRP-conjugated rabbit anti-human IgG, (Dako Ltd.) at 1/5000 dilution in blocking buffer before final washing and detection with H_2_O_2_ and O-phenylenediamine (Sigma). The reaction was stopped with 25μL of 2M H_2_SO_4_ per well and absorbance read at 492nm. The same positive controls (hyper-immune sera) were run in duplicate on each plate to allow for standardization of plate-to-plate variation. As high background reactivity was seen with non-malaria exposed sera (or UK sera) in results using 3D7 lysate, final results were obtained using A4 lysates (ELISA ODs to A4 and 3D7 lysates highly correlated; *p=<0.0001. r=0.8802*).

**Ethical & Regulatory Approval**

Ethical approval was granted by the ERC (KEMRI/RES/7/3/1) and the Oxford Tropical Research Ethics Committee (OXTREC 161-12). Regulatory approval was provided by the Kenyan Pharmacy and Poisons Board (PPB/ECCT/12/12/01/2013). The study was reviewed and allowed to proceed by the US Food and Drug Administration under IND 14267. The Safety Monitoring Committee (SMC) provided safety oversight and GCP compliance was independently monitored by the Clinical Trials Facility, Monitoring Department, Kenya Medical Research Institute and Wellcome Trust (Centre for Geographical Medicine Research – Coast), Kilifi, Kenya.

**PfSPZ Challenge Manufacture & Preparation for Administration**

PfSPZ Challenge was manufactured according to Good Manufacturing Practice (GMP) standards by Sanaria Inc. Manufacture includes the production, under traditional environmental conditions, of eggs from a colony of *A. stephensi* mosquitoes housed in a controlled environmental chamber. Surface disinfection of the eggs is performed by exposure to chemical agents in a Class II biosafety cabinet (BSC). From this point forward, all materials and product are handled using aseptic methods to ensure that contaminating microorganisms are not introduced to and carried through the process. Surface-disinfected eggs are inoculated into sterile, vented flasks containing aseptic growth medium. The eggs hatch and develop into pupae, which are transferred to an adult mosquito container where the adult mosquitoes emerge. These adult mosquitoes, which have been raised under aseptic conditions, are fed *P. falciparum* gametocyte-infected blood in a BSC in a High-Security Insectary in Rockville, Maryland, USA. The *P. falciparum* gametocyte-infected blood has been produced from cultures of the *P. falciparum* strain NF54 derived from a Master Cell Bank of the well-characterized *P. falciparum* strain NF54. Infected adult mosquitoes are maintained under aseptic conditions until *P. falciparum* sporozoites migrate to the salivary glands. The salivary glands from the *P. falciparum* sporozoite-infected mosquitoes are removed by hand dissection. Salivary glands are then triturated to release the *P. falciparum* sporozoites. The sporozoites are purified, counted, and, at a specified concentration, cryopreserved. Cryopreservation commences with the addition of cryoprotective additives to the purified sporozoites to produce the PfSPZ Challenge product. PfSPZ Challenge is dispensed into screw-cap vials containing 15,000 or 50,000 sporozoites in 20 µL aliquots. PfSPZ Challenge is stored in liquid nitrogen vapour phase (LNVP) at -150°C to -196°C.

Vials of PfSPZ Challenge were stored and transported to site in LNVP. Immediately prior to use, PfSPZ Challenge in cryovials was thawed individually by partial submersion of the vials for 30 seconds in a 37°C ± 1°C water bath. Designated, trained study staff then prepared, diluted (using Phosphate buffered saline (PBS) and 25% Human Albumin Solution (HSA) and dispensed PfSPZ Challenge to clinical staff at the site. The maximum time interval allowed between thawing of PfSPZ Challenge and administration to a volunteer was 30 minutes. If PfSPZ Challenge had not been administered within this time, the preparation was discarded and a fresh vial of PfSPZ Challenge was thawed and prepared for administration. Information regarding PfSPZ Challenge potency and viability overtime is included in table S4 (PfSPZ Challenge was 10 months old at the time of the trial).

**Administration of PfSPZ Challenge**

Volunteers were admitted and reviewed the day before CHMI (dC-1) to check on-going eligibility and blood drawn for safety assessment and baseline immunology. PfSPZ Challenge was administered using a needle and syringe intramuscularly (IM) using the BD 1mL syringe (Ref 309628) and BD Microlance 3 (23G 1”, 0.6 x 25mm Ref 300800). Study staff administrating PfSPZ Challenge wore gloves and eye protection. During administration of PfSPZ Challenge, advanced life support drugs and resuscitation equipment were immediately available for the management of anaphylaxis. For each group the total dose of sporozoites was divided into two equal doses and administered as 50 µL injections, one in each deltoid IM. The inoculation sites were immediately covered with a dressing to absorb any PfSPZ Challenge that may have leaked out through the needle track. The dressing was removed from the injection site after 1 hour when the volunteers were reviewed and provided with an emergency contact card detailing the 24-hour telephone number of the on-call study clinician and the sensitivities of the infecting strain of malaria.

Participants in Groups 1 and 2 were enrolled first on the same day. In the absence of safety concerns in these participants and following SMC review, 48 hours later the participants in Groups 3 and 4 were enrolled. In the absence of safety concerns and following SMC review, 120 hours later, 2 participants from Group 5 and 2 participants from Group 6 were enrolled. 48 hours later, in the absence of safety concerns and following SMC review, the remaining participants in Groups 5 and 6 were enrolled.

**Safety**

All AEs were graded according to the criteria in Tables S1 & S2. Volunteers were admitted as in-patients from the day before administration of PfSPZ Challenge until completion of anti-malarial therapy. All volunteers underwent once daily clinical review on days 1-5 post CHMI (dC+1-5) to enquire as to adverse events (AEs) and use of medications. Volunteers were reviewed on dC+6 in the evening and then twice a day, morning and evening between dC+7 and dC+14. Undiagnosed volunteers were reviewed once a day in the morning between dC+15 and C+21. At each visit, blood was sampled for microscopy and qPCR, physical observations performed and AEs solicited. Volunteers were treated with a 3-day curative course of oral Co-Artem®, where each dose was directly observed. Volunteers were reviewed 24 and 48 hours post-diagnosis where blood was sampled for microscopy and qPCR. Provided these two blood-films after treatment were negative for parasites, volunteers were not reviewed again in clinic until dC+35. If one of these blood films was positive, volunteers continued to be reviewed at 24 hour intervals until two consecutive blood films were negative. Volunteers were then reviewed at dC+35 and dC+90 where safety assessments were performed. Complete blood count with differential, platelet count and serum biochemistry (including electrolytes, urea, creatinine, bilirubin, alanine aminotransferase, alkaline phosphatase and albumin) were measured at screening, dC-1, dC+9, within 24 hours of diagnosis, and at dC+35 and dC+90. Blood was drawn for immunology at dC-1, dC+7, d+C9, d+C11, d+C13, d+C15, d+C17, d+C19 and d+C21 if persistently undiagnosed with malaria. Blood was then drawn for immunology on reviews in clinic on dC+35 and dC+90. Throughout the paper, study day refers to the nominal time-point for a group and not the actual day of sampling. Both laboratory personnel and microscopists were blinded to group allocation.

**Microscopy**

Blood smears were prepared and interpreted according to the “Consensus SOP for Malaria Microscopy in the Context of Clinical Challenge Trials.”([Laurens et al., 2012](#_ENREF_3)) Briefly, slides were prepared using 10μL of whole blood spread over a rectangle measuring 1 x 2 cm. Slides were stained using Giemsa stain solution (10% for slides from symptomatic volunteers, 4% for all other slides). Using a microscope with a high-power field of 0.22 mm diameter, 5 passes were read for samples from asymptomatic and treated volunteers and 10 passes for samples from symptomatic volunteers. A positive thick film on microscopy was defined as at least two morphologically normal, ‘unambiguous’ malaria parasites seen in 0.5μL of blood by two or more experienced microscopists blinded to each other’s findings. A parasite was considered ‘unambiguous’ if all features (including purple red chromatin dot, blue cytoplasm, dark mass of pigment and distinct morphology) were clearly visible. A parasite was considered ‘ambiguous’ if it did not demonstrate the features described above. All ‘positive’ slides were quantified for parasite density (parasites/µL). A third microscopist acted as slide coordinator, distributing the slides between microscopists and reconciling any discrepant findings. All microscopists working on the study passed a 2 week refresher-training course at the KEMRI-affiliated Malaria Diagnostics Centre of Excellence, Kisumu, Kenya approximately 3 months prior to working on study samples.

**qPCR and Parasite Growth Modelling**

For quality control purposes, qPCR samples were re-tested if;

- Replicates included a mixture of positive and negative (in terms of amplification) results with one or more positive results > 100 parasites/mL.
- The %CV of any results were high outliers.

In the following circumstances, the qPCR value was replaced with 5 parasites/mL;

- Any qPCR negative time-points immediately followed by qPCR positive time-point.
- qPCR values <5 parasites/mL.

Negative qPCR results seen 2 or more time-points before a qPCR positive result were listed as missing.

Simple linear regression was used to estimate the number of parasites released from the liver (liver-to-blood inoculum; LBI; parasite density at day 6.5 post administration of PfSPZ Challenge) and fold-change in parasites over 48 hours (parasite multiplication rate; PMR).([Douglas et al., 2013](#_ENREF_2)) Modelling was conducted using Stata version 11 (StataCorp, Texas). Time in hours between injection of PfSPZ Challenge and collection of each blood-sample was calculated using data specific to each volunteer for each of these events and then converted to days. PMR was only calculated for volunteers with 5 or more positive qPCR values. LBI was adjusted to account of volunteers’ individual blood volumes (blood volume = 70mL/kg).

**SUPPLEMENTARY REFERENCES**

Chong, S.S., Boehm, C.D., Cutting, G.R., Higgs, D.R., 2000. Simplified multiplex-PCR diagnosis of common southeast asian deletional determinants of alpha-thalassemia. Clinical chemistry 46, 1692-1695.

Douglas, A.D., Edwards, N.J., Duncan, C.J., Thompson, F.M., Sheehy, S.H., O'Hara, G.A., Anagnostou, N., Walther, M., Webster, D.P., Dunachie, S.J., Porter, D.W., Andrews, L., Gilbert, S.C., Draper, S.J., Hill, A.V., Bejon, P., 2013. Comparison of Modeling Methods to Determine Liver-to-blood Inocula and Parasite Multiplication Rates During Controlled Human Malaria Infection. The Journal of infectious diseases 208, 340-345.

Laurens, M.B., Duncan, C.J., Epstein, J.E., Hill, A.V., Komisar, J.L., Lyke, K.E., Ockenhouse, C.F., Richie, T.L., Roestenberg, M., Sauerwein, R.W., Spring, M.D., Talley, A.K., Moorthy, V.S., 2012. A consultation on the optimization of controlled human malaria infection by mosquito bite for evaluation of candidate malaria vaccines. Vaccine 30, 5302-5304.

Ndungu, F.M., Bull, P.C., Ross, A., Lowe, B.S., Kabiru, E., Marsh, K., 2002. Naturally acquired immunoglobulin (Ig)G subclass antibodies to crude asexual Plasmodium falciparum lysates: evidence for association with protection for IgG1 and disease for IgG2. Parasite immunology 24, 77-82.

Taylor, R.R., Smith, D.B., Robinson, V.J., McBride, J.S., Riley, E.M., 1995. Human antibody response to Plasmodium falciparum merozoite surface protein 2 is serogroup specific and predominantly of the immunoglobulin G3 subclass. Infection and immunity 63, 4382-4388.

Waterfall, C.M., Cobb, B.D., 2001. Single tube genotyping of sickle cell anaemia using PCR-based SNP analysis. Nucleic acids research 29, E119.

Williams, T.N., Wambua, S., Uyoga, S., Macharia, A., Mwacharo, J.K., Newton, C.R., Maitland, K., 2005. Both heterozygous and homozygous alpha+ thalassemias protect against severe and fatal Plasmodium falciparum malaria on the coast of Kenya. Blood 106, 368-371.

**SUPPLEMENTARY TABLES**

**Table S1: Criteria for Grading Severity of Local Adverse Events Related to PfSPZ Challenge Injection.**

* ≤3mm erythema was considered consistent with needle puncture.

| **Adverse Event** | **Grade** | **Intensity** |
| --- | --- | --- |
| Pain at injection site | 1 | Pain that is easily tolerated |
|  | 2 | Pain that interferes with daily activity |
|  | 3 | Pain that prevents daily activity |
| Erythema at injection site* | 1 | >3 - ≤50 mm |
|  | 2 | >50 - ≤100 mm |
|  | 3 | >100 mm |
| Swelling at injection site | 1 | >0 - ≤20 mm |
|  | 2 | >20 - ≤50 mm |
|  | 3 | >50 mm |

**Table S2: Functional Criteria for Grading Severity of Systemic Adverse Events.**

| **GRADE 0** | None |
| --- | --- |
| **GRADE 1** | Mild: Transient or mild discomfort (< 48 hours); no medical intervention/therapy required |
| **GRADE 2** | Moderate: Mild to moderate limitation in activity - some assistance may be needed; no or minimal medical intervention/therapy required |
| **GRADE 3** | Severe: Marked limitation in activity, some assistance usually required; medical intervention/therapy required, hospitalisation possible |

**Table S3. Demographics of Enrolled Volunteers.**

|  | **25,000** | **75,000** | **125,000** |
| --- | --- | --- | --- |
|  | **n = 4** | **n = 4** | **n = 20** |
| ***Sex*** | | | |
| Male | 2 (50%) | 4 (100%) | 11 (55%) |
| Female | 2 (50%) | 0 | 9 (45%) |
| ***Age at screening (years)*** | | | |
| Mean (SD) | 24 (1.6) | 23 (2.5) | 26 (2.9) |
| Median | 24 | 23 | 26 |
| Min, Max | 22, 26 | 19,25 | 22,31 |

**Table S4. Results of potency and sporozoite membrane integrity assays on the lot of PfSPZ Challenge (Lot071112-02 (PC50-02)) used in the KEMRI Challenge Study**

| **Time Point** | **Potency**  **(number of parasites expressing**  **PfMSP-1/well)** | **% Viability**  **(sporozoite membrane integrity assay)** |
| --- | --- | --- |
| Fresh | 28.3 ± 1.5 parasites | 95.5% |
| Release | 25.3 ± 1.5 parasites | 89% ± 2.2% |
| 3 Month | 21.7 ± 1.5 parasites | 85% ± 3.3% |
| 6 Month | 25.0 ± 5.3 parasites | 86.2% ± 4.8% |
| 9 Month | 19.0 ± 1.0 parasites | 85.2% ± 4.4% |
| 12 Month | 19.3 ± 1.2 parasites | 83.1% ± 2.5% |

**Table S5: End Points for Treatment of Subjects.** BF = blood film.

|  | **25,000**  **n=4** | **75,000**  **n=4** | **125,000**  **n=20** |
| --- | --- | --- | --- |
| Symptomatic with positive BF | 0 | 0 | 5 (25%) |
| Symptomatic with negative BF | 0 | 0 | 0 |
| Asymptomatic with positive BF | 3 (75%) | 4 (100%) | 15 (75%) |
| Reached Day 21 undiagnosed (asymptomatic, BF negative) | 1 (25%) | 0 | 0 |
| Withdrew Consent or Excluded From Study Prior to Primary Endpoint | 0 | 0 | 0 |

**Table S6: Summary of Study Information.**

|  | **Group 1 (n=2)** | **Group 2 (n=2)** | **Group 3 (n=2)** | **Group 4**  **(n=2)** | **Group 5**  **(n=10)** | **Group 6**  **(n=10)** |
| --- | --- | --- | --- | --- | --- | --- |
| Dose (number of PfSPZ & volume) | 25,000 IM 50ul | 25,000 IM 50ul | 75,000 IM 50ul | 75,000 IM 50ul | 125,000 IM 50ul | 125,000 IM 50ul |
| Number volunteers thick smear positive (TS+) | 2 | 1 | 2 | 2 | 10 | 10 |
| Listing of times to TS+ (days) | 12.3, 12.8 | 11.9 | 11.2, 12.3 | 12.2, 14.3 | 12.3, 12.3, 12.1, 10.9, 12.4, 11.8, 11.2, 10.8, 11.2, 12.8 | 11.9, 10.8, 11.8, 12.2, 14.3, 11.1, 13.8, 13.1, 11.8, 11.8 |
| Listing of parasite density at time of TS+ (parasite/μl blood) | 7,4 | 7 | 5,6 | 8,5 | 6,6,4,5,8,5,5,5,5,7 | 6,6,5,6,7,6,6,7,7,9 |
| Geometric mean parasite density at time of TS+ (parasites/μl) | 5.3 | 7 | 5.5 | 6.3 | 5.5 | 6.4 |

**Table S7: Adverse Events Possibly, Probably or Definitely Related to Clinical *P. falciparum* infection According to Group.** % = percentage of volunteers affected. ALT = alanine transaminsase.

| **System Organ Class** | **Adverse Event** | **Group 1** | **Group 2** | **Group 3** | **Group 4** | **Group 5** | **Group 6** | **Total n=28 (%)** |
| --- | --- | --- | --- | --- | --- | --- | --- | --- |
|  |  | **n=2 (%)** | **n=2 (%)** | **n=2 (%)** | **n=2 (%)** | **n=12 (%)** | **n=12 (%)** |  |
| Blood and lymphatic system disorders | Lymphopenia | 1 (50) | 0 (0) | 0 (0) | 1 (50) | 5 (42) | 5 (42) | 12 (43) |
|  | Neutropenia | 0 (0) | 0 (0) | 0 (0) | 0 (0) | 1 (8) | 1 (8) | 2 (7) |
|  | Leucopenia | 0 (0) | 0 (0) | 0 (0) | 0 (0) | 2 (17) | 0 (0) | 2 (7) |
|  | Thrombocytopenia | 0 (0) | 0 (0) | 0 (0) | 0 (0) | 0 (0) | 1 (8) | 1 (4) |
|  |  |  |  |  |  |  |  |  |
| Cardiac disorders | Tachycardia | 2 (100) | 0 (0) | 0 (0) | 0 (0) | 2 (17) | 2 (17) | 6 (21) |
|  |  |  |  |  |  |  |  |  |
| Gastrointestinal disorders | Abdominal Pain | 2 (100) | 1 (50) | 0 (0) | 0 (0) | 2 (17) | 1 (8) | 6 (21) |
|  | Nausea | 1 (50) | 1 (50) | 1 (50) | 0 (0) | 3 (25) | 1 (8) | 7 (25) |
|  | Vomiting | 1 (50) | 1 (50) | 0 (0) | 0 (0) | 0 (0) | 1 (8) | 3 (14) |
|  | Anorexia | 1 (50) | 1 (50) | 1 (50) | 0 (0) | 6 (42) | 3 (25) | 12 (43) |
|  | Diarrhoea | 1 (50) | 0 (0) | 0 (0) | 0 (0) | 0 (0) | 2 (17) | 3 (11) |
|  |  |  |  |  |  |  |  |  |
| General disorders | Malaise | 0 (0) | 1 (50) | 0 (0) | 0 (0) | 1 (8) | 0 (0) | 2 (7) |
|  | Chills | 2 (100) | 1 (50) | 0 (0) | 0 (0) | 5 (42) | 3 (25) | 11 (39) |
|  | Fatigue | 2 (100) | 1 (50) | 2 (100) | 1 (50) | 4 (33) | 5 (42) | 15 (54) |
|  | Sweating | 2 (100) | 0 (0) | 0 (0) | 0 (0) | 6 (50) | 4 (33) | 12 (43) |
|  | Rigor | 2 (100) | 0 (0) | 0 (0) | 0 (0) | 3 (25) | 1 (8) | 6 (21) |
|  | Fever | 2 (100) | 2 (100) | 0 (0) | 1 (50) | 9 (75) | 8 (67) | 22 (79) |
|  |  |  |  |  |  |  |  |  |
| Hepatobiliary | Elevated ALT | 0 (0) | 0 (0) | 0 (0) | 0 (0) | 0 (0) | 1 (8) | 1 (4) |
|  |  |  |  |  |  |  |  |  |
| Musculoskeletal and connective tissue disorders | Arthralgia | 1 (50) | 1 (50) | 0 (0) | 2 (100) | 5 (42) | 5 (42) | 14 (50) |
|  | Myalgia | 1 (50) | 0 (0) | 0 (0) | 1 (50) | 6 (50) | 5 (42) | 13 (46) |
|  | Back Pain | 1 (50) | 0 (0) | 0 (0) | 1 (50) | 5 (42) | 5 (42) | 12 (43) |
|  | Neck Pain | 1 (50) | 0 (0) | 0 (0) | 0 (0) | 1 (8) | 0 (0) | 2 (7) |
|  |  |  |  |  |  |  |  |  |
| Nervous system disorders | Headache | 2 (100) | 1 (50) | 2 (100) | 1 (50) | 10 (83) | 9 (75) | 25 (89) |
|  | Migraine | 0 (0) | 0 (0) | 0 (0) | 0 (0) | 0 (0) | 0 (0) | 0 (0) |
|  |  |  |  |  |  |  |  |  |
| Respiratory, thoracic and mediastinal disorders | Epistaxis | 1 (50) | 0 (0) | 0 (0) | 0 (0) | 0 (0) | 0 (0) | 1 (4) |
|  |  |  |  |  |  |  |  |  |

**Table S8: Adverse Event Data Possibly, Probably or Definitely Related to Clinical *P. falciparum* infection According to Severity.** % = percentage of volunteers affected. ALT = alanine transaminsase.

| **System Organ Class** | **Adverse Event** | **Severity Grading** | | | **Total** |
| --- | --- | --- | --- | --- | --- |
|  |  | **Mild** | **Moderate** | **Severe** | **n=28** |
|  |  | (%) | (%) | (%) | (%) |
| Blood and lymphatic system disorders | Lymphopenia | 10 (36) | 2 (7) | 0 (0) | 12 (43) |
|  | Neutropenia | 2 (7) | 0 (0) | 0 (0) | 2 (7) |
|  | Leukopenia | 2 (7) | 0 (0) | 0 (0) | 2 (7) |
|  | Thrombocytopenia | 1 (4) | 0 (0) | 0 (0) | 1 (4) |
|  |  |  |  |  |  |
| Cardiac disorders | Tachycardia | 3 (11) | 2 (7) | 1 (4) | 6 (21) |
|  |  |  |  |  |  |
| Gastrointestinal disorders | Abdominal pain | 2 (7) | 4 (14) | 0 (0) | 6 (21) |
|  | Nausea | 3 (11) | 3 (11) | 1 (4) | 7 (25) |
|  | Vomiting | 0 (0) | 0 (0) | 3 (11) | 3 (11) |
|  | Anorexia | 6 (21) | 6 (21) | 0 (0) | 12 (43) |
|  | Diarrhoea | 2 (7) | 1 (4) | 0 (0) | 3 (11) |
|  |  |  |  |  |  |
| General disorders and administration site conditions | Malaise | 0 (0) | 1 (4) | 1 (4) | 2 (7) |
|  | Chills | 4 (14) | 5 (18) | 2 (7) | 11 (39) |
|  | Fatigue | 7 (25) | 5 (18) | 3 (11) | 15 (54) |
|  | Sweating | 4 (14) | 4 (14) | 4 (14) | 12 (43) |
|  | Rigor | 0 (0) | 0 (0) | 6 (21) | 6 (21) |
|  | Fever | 9 (33) | 11(39) | 2 (7) | 22 (79) |
|  |  |  |  |  |  |
| Hepatobiliary | Elevated ALT | 1 (4) | 0 (0) | 0 (0) | 1 (4) |
|  |  |  |  |  |  |
| Musculoskeletal and connective tissue disorders | Arthralgia | 11 (39) | 3 (11) | 0 (0) | 14 (50) |
|  | Myalgia | 10 (36) | 3 (11) | 0 (0) | 13 (46) |
|  | Back pain | 6 (21) | 3 (11) | 3 (11) | 12 (43) |
|  | Neck pain | 1 (4) | 1 (4) | 0 (0) | 2 (7) |
|  |  |  |  |  |  |
| Nervous system disorders | Headache | 11 (39) | 9 (32) | 5 (18) | 25 (89) |
|  | Migraine | 0 (0) | 0 (0) | 0 (0) | 1 (4) |
|  |  |  |  |  |  |
| Respiratory, thoracic and mediastinal disorders | Epistaxis | 1 (4) | 0 (0) | 0 (0) | 1 (4) |
|  |  |  |  |  |  |
